# Post-Synapses in the Brain: Role of Dendritic and Spine Structures

**DOI:** 10.3390/biomedicines10081859

**Published:** 2022-08-02

**Authors:** Jacopo Meldolesi

**Affiliations:** 1 San Raffaele Institute, Vita-Salute San Raffaele University, 20132 Milan, Italy; meldolesi.jacopo@hsr.it; 2 CNR Institute of Neuroscience, Milan-Bicocca University, 20132 Milan, Italy

**Keywords:** post-synapse, dendrite, dendritic fiber, arborization, microdomain, cytoskeleton, flat/entended, spine

## Abstract

Brain synapses are neuronal structures of the greatest interest. For a long time, however, the knowledge about them was variable, and interest was mostly focused on their pre-synaptic portions, especially neurotransmitter release from axon terminals. In the present review interest is focused on post-synapses, the structures receiving and converting pre-synaptic messages. Upon further modulation, such messages are transferred to dendritic fibers. Dendrites are profoundly different from axons; they are shorter and of variable thickness. Their post-synapses are of two types. Those called flat/intended/aspines, integrated into dendritic fibers, are very frequent in inhibitory neurons. The spines, small and stemming protrusions, connected to dendritic fibers by their necks, are present in almost all excitatory neurons. Several structures and functions including the post-synaptic densities and associated proteins, the nanoscale mechanisms of compartmentalization, the cytoskeletons of actin and microtubules, are analogous in the two post-synaptic forms. However other properties, such as plasticity and its functions of learning and memory, are largely distinct. Several properties of spines, including emersion from dendritic fibers, growth, change in shape and decreases in size up to disappearance, are specific. Spinal heads correspond to largely independent signaling compartments. They are motile, their local signaling is fast, however transport through their thin necks is slow. When single spines are activated separately, their dendritic effects are often lacking; when multiple spines are activated concomitantly, their effects take place. Defects of post-synaptic responses, especially those of spines, take place in various brain diseases. Here alterations affecting symptoms and future therapy are shown to occur in neurodegenerative diseases and autism spectrum disorders.

## 1. Introduction

Brain synapses, main structures of neuronal interactions, operate by two subsequent processes. The first, activated by stimulation of pre-synaptic axon terminals, includes the synthesis and release of specific neurotransmitters; the second, triggered at post-synaptic structures of dendritic fibers, includes the activation of specific neurotransmitter receptors followed by their comprehensive transduction. Ensuing intracellular processes may then participate by widely integrated post-synaptic signaling [1].

Even if integration of pre-synapses and post-synapses was known, interest about them remained indifferent. For several decades knowledge of the first did grow, reaching very high levels, while knowledge of the second remained limited [1]. After 1990, however, interest in post-synapses began to increase, reaching a high level about 10 years ago [1,2,3]. It is therefore time to reconsider the state of post-synaptic processes, focusing especially on recent developments of relevant properties, including their heterogeneity and complexity.

Upon receiving their pre-synaptic messages, post-synaptic structures undergo processing, first by activation of factors associated to their post-synaptic densities (PSDs), including molecular machines directly connected to specific signaling frameworks [4]. In other words, post-synaptic PSDs operate as neuronal antennae, receiving and then converting signaling inputs, finally to adjacent dendritic fibers [4,5,6,7]. The complexity of such signaling does not depend only on the nature of the received pre-synaptic messages. It is also due to the complexity and functionality of the two types of post-synaptic structures, one located at flat or indented areas of dendritic fibers, the other at the spines, small expansions connected to dendritic fibers by their neck [8,9]. Additional types of post-synapses, located not at dendritic fibers but at neuronal bodies [1], will not be considered in the present review.

In the brain, the distribution of flat/intended and that of spinal post-synapses are not random. Coverage by flat/intended predominates in dendritic fibers of inhibitory neurons; coverage by spines (discovered by Santiago Ramon y Cajal at the end of the 19th century) accounts for almost all dendritic fibers from excitatory neurons [8]. Functionally the two types of post-synapses may appear largely analogous. However, in specific effects such as plasticity, relevant for processes including differentiation/de-differentiation, learning and memory, the flat-to-spine prominence is considerable [9,10,11]. Additional properties, such as structures, molecular composition, mechanisms, dynamics and time-dependence of their activity, are more relevant or specific of the spines [3,8,10,11,12].

In conclusion, the differences in the two types of post-synapses are of great relevance for brain structure and function. Here such differences are illustrated in two subsequent sections. Dendritic synapses of flat/indented/aspinal [13] structure are illustrated in a relatively short Section 1. This is because many important properties of these structures had been established in previous decades [1,2,3,4,5,6]. Section 2, dedicated to spine synapses, is longer because knowledge about these structures has grown considerably during the last several years [8,10,11,12], and recent developments have revealed important news and also a few aspects that at present are known only partially [8,11,13]. Finally, a Section 3 has been included dedicated to the role of post-synaptic processes in a few brain diseases, illustrating processes of relevance in which the two forms of post-synapses induce peculiar operational properties.

## 2. Flat/Intended/Aspine Post-Synapses

**Figure 1:**
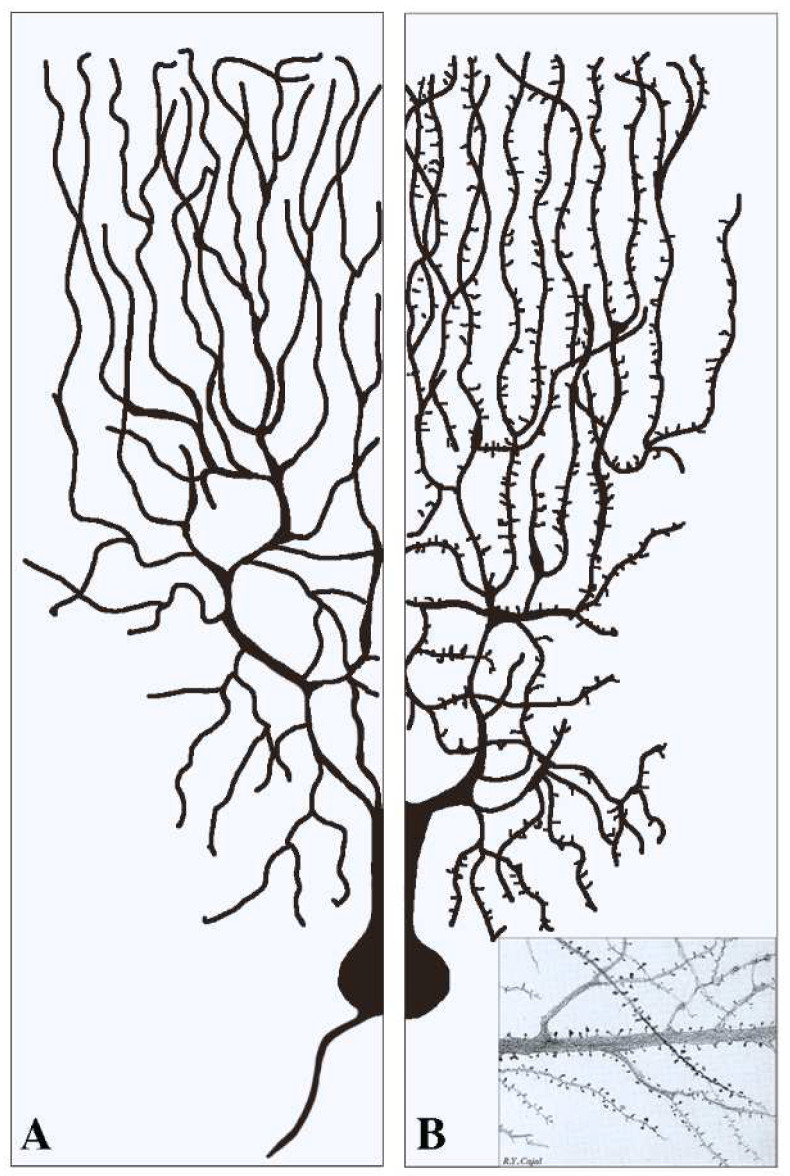
Examples of two dendritic arborizations with flat/intended (**A**) and spinal (**B**) post-synapses in brain neurons. In neurons the distribution of the dendritic fibers from the cell body is shown at a side opposite to that of axons (see (**A**)). Pre-synapses associated with dendritic post-synapses are not shown. In (**A**) all dendritic fibers appear smooth because their post-synapses, predominant in inhibitory neurons, are flat/intended, i.e., they do not emerge or emerge only marginally from the fiber surface. Dendritic fibers shown in (**B**), analogous in general shape to those in (**A**), predominate in stimulatory neurons. Beginning at some distance from the cell body, these fibers are covered by a high density of post-synapses composed by spines. The insertion in (**B**) is a fraction of an original figure by Santiago Ramon y Cajal (1896), reported as the CAT 024 figure in the book *Ciencia y Arte* by the Instituto Cajal, Madrid, 2004.

This section deals with the synapses established pre-synaptically by axonal terminals integrated with flat post-synapses inserted directly in the dendritic fibers. As already mentioned, these fibers, typically devoid of spines (Figure 1A), largely predominate in inhibitory neurons. Compared to axons, dendritic fibers differ in many respects [11,12]; they are shorter, and their cross sections are thicker and less uniform. Their frequent branching occurs at semi-regular intervals, with typical arbors, starting in the proximity of the cell body and distributed to the whole space. The intense branching of dendrites appears largely controlled by various forms of GTPase also involved in spine generation [2,12]. The flat/indented/aspine post-synapses include small fractions of organelles such as endoplasmic reticulum (ER), Golgi complex and endocytic system, less abundant than those of neuronal bodies. These organelles account for local functions including protein synthesis and transport, generated within or in their proximity [12,13,14,15]. Relevant to this organization and function are the cytoskeletons of both actin and microtubules [16,17]. In addition, post-synaptic areas are important for other functions, including regulation of ionic homeostasis [15].

### 2.1. Structural Properties of Dendritic Post-Synapses

The predominant structures of post-synapses, already mentioned in the Introduction [4,5], include the PSD machineries, composed by adhesion molecules, neurotransmitter receptors, enzymes, and associated scaffolding proteins. PSD is the first place where receptor activation/inhibition starts the signaling, leading to the post-synaptic function. For at least some of their effects, post-synaptic PSDs are compartmentalized into nanodomains, clustered for specialized proteins upon appropriate signaling [11,15,18].

Moreover, the dendritic synapses are regulated by cytoskeletons. Actin fibers, in addition to their distribution and function in dendritic shafts and branches, are spread within the dendritic synapses, with penetration up to the PSDs [17]. Intracellular binding of actin fibers contributes to the nature and structure of scaffolding proteins, and thus to the extracellular tethering of neurotransmitters to their specific receptors, a process of great relevance in the regulation of synaptic gating [17,19]. Both positive and negative controls of these effects are mediated by serum response factors (SRFs), abundant in the synaptic areas together with its MKL/MRTF cofactor [20]. Microtubules, operative from their specific cytoskeleton, are important for both the composition and orientation of dendritic fibers and synapses. At variance with the distal pointing of the plus end, typical of axons, the microtubules of dendrites are of mixed distribution [1,16].

### 2.2. Functions of Dendritic Post-Synapses

In the flat/intended/aspine dendritic post-synapses, the role of actin and microtubule cytoskeletons [16,17] depends on their interaction with receptors. Two types of ionotropic glutamatatergic receptors are included in the post-synaptic PSDs: NMDA, largely stable, and AMPA, widely distributed also in the extra-synaptic plasma membrane. In functional terms, AMPA receptors are involved in the trafficking of endocytic organelles taking place especially upon cell activation [21]. Various actin-associated proteins, such as cofilin, are involved in the establishment of protein interactions, including actin polymerization and NMDA regulation. The function of actin is not limited to excitatory receptors. In fact, actin filaments and their associated proteins operate also regulating inhibitory synapses, by acting on GABA and other receptors [19]. However, the activity of other inhibitory receptors, such as glycine and gephyrin receptors, does not depend on actin. Their direct regulation by microtubules induces their peculiar distribution on PSDs [16,19].

A critical function of post-synapses, largely dependent on dendrites, is plasticity, the capacity of cells to undergo changes including differentiation and de-differentiation. Such functions are related to main critical processes, such as learning and memory. Learning is a process involved in neural network transformations, made up by the synaptic connections of participating neurons; memory, established by reorganization of dendrite dynamics, consists in the maintenance of transformed networks. Both of these processes require structural and functional modifications of post-synapses [22,23].

In other post-synapses, however, the regulation of activity-dependent plasticity is different. In some excitatory synapses such activity depends on the brain-derived neurotrophic factor (BDNF), a neurotrophin critical also for the regulation of long-term potentiation (LTP) [24]. For the opposite process, long-term depression (LTD), recent results in mice have demonstrated the critical role of autophagy in dendritic synapses activated by glutamate receptors. In these experiments, inhibition of autophagy abolishes LTD and triggers neuronal LTP in the hippocampus [25].

Learning and memory of many complex functions of the brain are essential for animal survival [23]. Evidence of the last years has demonstrated the role of RNAs for the plasticity and many other functions. Non-coding, activity-dependent RNAs (ncRNAs), abundant in dendrites, are highly enriched at post-synapses where they appear critical for the plasticity of responses, including learning and memory [26]. Also critical for learning and memory of dendritic post-synapses are various microRNAs (miRs) involved in the regulated expression of numerous mRNAs involved in the translational synthesis of proteins [27]. The role of plasticity has remained unclear for decades. The advent of high-resolution time-lapse imaging, employed in conjunction with fluorescent biosensors and actuators, has enabled researchers to monitor and manipulate the structure and function of synapses, both in vitro and in vivo. The study of molecular signals associated with neuronal activities, including the activation of early genes, have led to the identification of the roles of RNAs in neuronal populations involved in memory coding [26,27].

### 2.3. Local Depolarizations and Action Potentials

The potentials produced at single dendritic post-synapses, no matter what their distance from the cell body, are too small to generate action potentials, which require summation of multiple synaptic potentials generated within short time intervals [1,2,28]. At first approximation, however, a dendritic branch resembles a leaky cable. In these conditions, therefore, synaptic potentials are expected to become smaller and smaller due to current leakage at multiple sites of branch organization. During the last decade the investigation of post-synapses has been strengthened, on the one hand, by probing the dendritic role of Ca2+ imaging [29]; on the other hand, by clarifying the involvement of various types of voltage-gated channels [29,30]. In dendrites the variable distance of synapses from the cell body is compensated by their size, increasing with distance from the cell body and by the ion gradients established along the dendrites [28,31].

## 3. Post-Synapses with Spines

**Figure 2:**
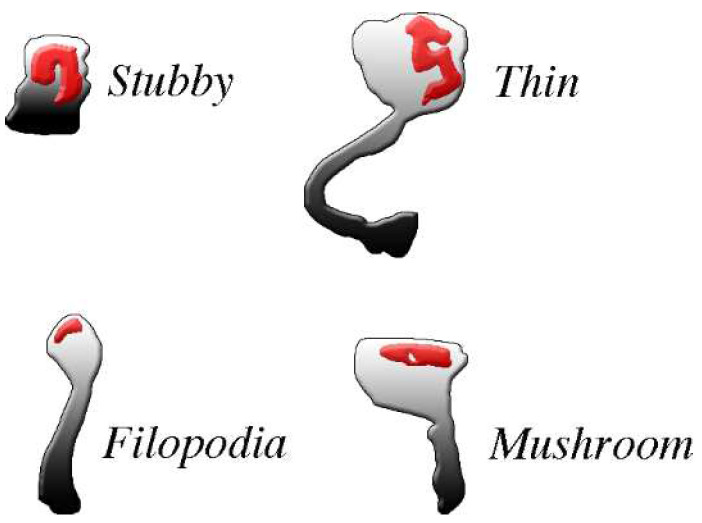
The four common types of post-synaptic spines are clearly different from each other. The density and shape of post-synaptic spines, abundant in excitatory neurons, change profoundly during physiological and pharmacological events. For example, changes occur during spine generation, by continuous turnover with regeneration, and by conversion of one type of spine into another. All spines exhibit abundance of PSD (red) distributed within the body in the proximity of the plasma membrane where pre-synaptic messages are received. PSDs are widely composed by adhesion molecules bound by scattered receptors, enzymes and at least some scaffolding proteins. Most PSD-bound proteins are critical for post-synaptic responses. Spines exhibit distinct shapes: stubbles do not have long necks, thus their responses are similar to those of the flat post-synapses; filopodia, often active in groups, are long but thin, with very small heads; mushroom and thin spines exhibit relative large heads, with flat and round top surfaces, respectively, connected to their dendritic fibers by long or very long necks. Their activities tend therefore to operate independently, with limited interactions with their dendritic fiber. Permission for this Figure, a fraction of Figure 2 of [36], has been obtained from Frontiers in Neuroscience.

The spines, small protrusions stemming from dendritic shafts, are the second type of post-synaptic structures activated by signals delivered by pre-synaptic transmission. As already mentioned, the presence of spines, typical of dendritic fibers, is appreciable at some distance from the cell body of most excitatory neurons (Figure 1). In inhibitory neurons the spines account for only a small fraction (about 10%) of the post-synapses, except for GABAergic neurons, in which the percentage is higher [32,33]. All spines exhibit dynamic forms, dependent on the received excitatory and inhibitory inputs. The structure of most established spines is recognized according to four morphological categories, critical for normal brain functions: filopodia, stubby, thin, and mushroom (Figure 2), generated separately or by switching from each other in a variety of conditions [34,35]. 

In functional terms the spines are not merely tunable memory elements. They are also embody algorithms that implement the brain’s ability to learn from pre-synaptic experience and cope with new challenges. Their forms, modulated downstream, depend on space-time events operative not at whole spines, but at spine microdomains [37]. A recent study has demonstrated a kind of synaptic signaling taking place not only from pre- to post-synaptic structures, but also backwards. Mechanical enlargement of the spines, induced by evoked release of excitatory neurotransmitters such as glutamate, can in fact proceed by pushing across the synaptic cleft, inducing stimulation of the pre-synaptic plasma membrane [38].

Spine assembly has been shown to depend on drebrin, a protein destined to become spine-resident upon its binding to both F actin of the cytoskeleton and PSD-95 of post-synaptic density [39,40,41]. During nascent spine formation actin undergoes reorganization. Specifically, its dynamic assembly/disassembly participates in the generation of microdomains; its interaction with specific cytoskeletal and membrane components supports modulation of synaptic functions [35,41]. The density of spiny post-synapse distribution, growing in children, reaches high values in mature humans, when density of inhibitory synapses is lower [42,43]. During their existence, many spines undergo dynamic changes, going from appearing, to enlarging, shrinking, and disappearing. When dependent on excitatory/inhibitory inputs and experience, their activities are defined extrinsic; when independent of external inputs, they are defined intrinsic [43,44]. Intrinsic dynamics play important roles in memory management and adaptations [35].

### 3.1. Structure and Composition of Spines

Generation of spines is due to several processes of relevance. Confocal microscopy studies have identified the mechanisms by which spines emerge from dendrite fibers and their individual structure is recognized. The mechanisms by which various types of spines initiate their activity are well known [35,40,43]. The ensuing processes are highly relevant. The first, discovered two decades ago, deals with the assembly of the so-called spine apparatus, a structure composed by stacks of smooth ER interdigitated by electron-dense plates composed primarily by proteins [45,46]. Generation of the spine apparatus requires the presence of synaptopodin, a 100 kDa actin-binding protein, concentrated on the spine neck. In the absence of the synaptopodin the apparatus does not assemble, and this precludes the formation of the spine. The functions of synaptopodin and the spine apparatus are multiple. Among them is the intracellular release of Ca2+ from the ER, necessary for synaptic plasticity to occur [45,46,47,48]. The role of synaptopodin and spine apparatus in the physiology of spines, particularly in the enlargement and clustering of spines, and in the cell establishment of LTP, have been confirmed by recent studies [13,49].

Super-resolution microscopy investigation, developed recently in living neurons, has revealed the regulation of synaptic structures by nanoscale mechanisms [35,50,51]. Their compositional properties are analogous to those already reported in flat/intended post-synapses. They include PSD complexes of adhesion proteins (Figure 2), including spaces occupied by receptors, enzymes, and scaffolding proteins [11,18]. The ensuing effects exhibit various levels of distinction [52]. Spine morphogenesis is largely regulated by actin cytoskeleton and annexin A1 [34,42]. In vivo studies have demonstrated that spines are motile: their plastic structures are influenced by sensory changes [51]; and their remodeling is established upon a variety of physiological stimuli [52,53,54]. In view of their narrow neck, many forms of spinal head correspond to largely independent signaling compartments (Figure 1). In other words, signaling of spines is fast locally, although its transport through long necks is slow and also inefficient [53,54]. Energy necessary to support spine function, provided by GTPases, is considerable [55,56]. The spine apparatuses, including in their cytoplasm small protrusions of ER transported and then stabilized via the actin-based motor myosin V, are in some cases analogous to structures of flat/endented post-synapses. Inclusion of ER contributes to the local Ca2+ homeostasis, up to the control of the spines’ strength [57,58].

An additional property of spines deals with their surface. As discovered over a decade ago, their post-synaptic membrane is often structured into multiple, spatially delimited microdomains [59,60,61]. Such heterogeneous structure allows the spines to execute separate functions together with electrical signals, relevant for synaptic transmissions, mediated by fast ligand-gated receptors, both of ionotropic and GTP-dependent structure [18,42].

As a whole, however, the structure of spinal post-synapses is not stable but continuously adjusted. The mechanisms of such adjustments, based on the present knowledge of molecular spine regulation, depend on the reorganization of the actin cytoskeleton and its sub-synaptic structures [16,42]. Interesting results in this area have been obtained by comparing the differences of post-synapses occurring in mice after sleep and appropriate stimulation. After sleep, clusters of nearly inactive filopodia spines are appreciable in dendrites; upon stimulation, new single spines appear largely different from filopodia [62]. The spiny structure, however, does not depend only on the dynamics of their changes. New establishments and ensuing stabilizations take place also in response to specific neuronal components of relevant function, such the tyrosine kinase Pyk2 and KIF5C, a member of the kinesin superfamily of molecular motors [63,64]. Interestingly, regulation of post-synapses, in particular those with spines, is controlled not only by neurons, but also by microglia. Upon depletion of these cells the synapses have been shown to reduce their spontaneous and evoked glutamatergic activity of the mouse hippocampus, associated to decreased expression of spines. In the adult brain, therefore, microglia contribute to normal synaptic function [65].

### 3.2. Function of Spines

The studies reported in this subsection should be considered as the prolongation of those about structure and composition of spines, already reported in Section 3.1. The latter studies were related particularly to physiology [7,41,47,50] and plasticity of induced processes, such as learning and memory [9,10,11,24,25,37,40,42,55,56]. In the present subsection, the function of these processes is considered from a general point of view.

The key relevance of Ca2+ in the rapid control of neuronal functions, based on the involvement of ryanodine and IP3 receptors followed by activation of SERCA pumps, has been known for decades [66]; recent developments have clarified the interactions among processes occurring in dendritic spines. Release of Ca2+ from the ER is followed by a rapid replenishment now shown to depend on two properties of the system: the small amplitude and slow timescale for the Ca2+ influx via surface channels, and the close proximity of the spine apparatus with the plasma membrane. Thus, the spine organization of Ca2+ also determines their Ca2+ dynamics [67,68]. Such a state is relevant especially in the spines where the diffusion of ions and molecules is markedly slowed down by the curvature of its connections taking place at the heads and the necks [69].

The functions of spines, illustrated on spatial and temporal scales, can be envisaged in terms of plasticity. The latter is connected to several processes, including learning and memory, controlled by structural and functional modifications of synaptic connections. A variety of approaches, based on pharmacological, genetic, molecular, imaging, and optical technologies, have been developed and applied to dissect the complexity of signal transduction pathways. The combination of conventional and new methodological approaches has led to the identification and manipulation of key molecular mechanisms related to spine plasticity and regulation, starting from molecular events occurring in single spines [52,55,56]. The activity of each protein involved displays specific space-temporal patterns, coordinating the downstream events at different micro-domains, thus changing the morphology and function of spines [51,70,71]. Extension of the approaches to various neuronal networks could induce information of spine processing at different brain areas [71].

Formation of long-term memory creates long-lasting changes. Learning-induced changes in the spine can contribute to the formation of neuronal networks of memory. Specifically, the learning-induced spine modifications can result in changes in post-synaptic functions, generating and maintaining long-term memory within neuronal networks [42,70,71]. Essential mechanisms active in spine generation and plasticity depend on actin cytoskeleton, a highly dynamic structure. In addition to the spine apparatus, the long-lasting function of actin cytoskeleton depends on the local concentration and properties of various regulatory and scaffold proteins [71,72,73]. Tyrosine phosphorylation rapidly induces changes in actin function, with increased dynamics and fast reorganization of the cytoskeleton [74]. In spines, therefore, actin cytoskeleton supports not only structure but also brain memory.

The functional processes of spines are mostly governed by regulatory mechanisms. Present evidence favors small GTPases. Specifically, members of the Rho family have been shown to remodel the actin cytoskeleton by affecting various downstream molecules, thus helping to reveal the mechanisms by which memory is formed [75]. The involvement and the role of GTPases have been confirmed by results with GS14, a multifunctional signaling protein regulator of synaptic plasticity. Such protein participates also in the transport of secretory proteins from the nucleus to the cytoplasm, with accumulation in the spines [76]. Numerous functions regulated by GS14 in the spines include the role of various G proteins and Ca2+/calmodulin signaling [76]. Another study has demonstrated the increased number and activity of spines, reduced in a mouse family affected by an Alzheimer’s disease model, reaches values of normal mice after months of treadmill exercise [77]. It appears, therefore, that brain functions, including learning and memory, can be protected at the level of spines by appropriate physical exercise.

### 3.3. Spine Depolarizations and Voltage Gradients

Electric inputs to spines have been investigated over the last two decades: or years, however, the results obtained have been questioned. Optical imaging has revealed the formation of spine foci receiving locally convergent inputs from pre-synaptic cell assemblies. Such form of clustered synaptic plasticity was proposed to integrate events along dendrites [78]. In other studies, single spine activations have been found completely ineffective for most excitatory synapses due to the distorted recruitment of voltage-dependent channels. The concomitant large AMPA conductance produces saturation of spine responses, affecting appropriate effects of synaptic currents [79]. In the CNS the spines mediate most excitatory neurotransmissions. Spines, although biochemically compartmentalized, modify electrical synaptic potentials. To investigate the problem, the voltage imaging data were combined with electro-diffusing modeling. The results demonstrated that the post-synaptic voltage depends also on the spine geometry. A key property of spines, their variability in terms of size and shape, play critical roles in determining synaptic transduction [80]. In those conditions, spines often experience voltages different from those of parent dendrites, thus appearing of independent activation. Spines, therefore, are elementary voltage compartments, important for synaptic function and plasticity, relevant for dendritic integrations and also for disease states [81]. Electro-diffusion can be used to interpret voltage distribution in neuronal microdomains. In various dendrites, synaptic density changes in response to specific actions. For example, changes in dendritic receptors could operate in the maintenance of spines in cholinergic interneurons, activating mechanisms operative in Parkinson’s disease [82].

## 4. Post-Synapse Alterations in Brain Diseases

This section is not entirely distinct from the previous ones. Indeed, many studies about structure and function of post-synapses, especially those concerning the spines, include sections dealing with diseases; see for example [16,25,53,73,75,77]. The difference between the previously mentioned articles and those of this section deals not with different areas but with different approaches, the most important in the present section. Another important property of this section depends on the various ages of patients. In particular, cellular senescence grows interplayed to plasticity, with ensuing limitations of core mechanisms such as learning, memory and cognition [83,84,85]. In the brain, aging defects depend largely on the decreased number and maturity of spines and decreased expression of glutamate receptors, ultimately leading to increasing defect of basic functions [85]. In many neurological and neuropsychiatric diseases, the pathological deficits are based on the disruption of spine functions with impairment in neuronal connectivity and plasticity. These dysregulations are usually accompanied by morphological alterations to the spine number, shape and size occurring at early pathological stages, often before the appearance of clinical manifestations [86]. However, spine disruptions play critical roles in numerous brain diseases. Here I introduce results concerning only a few of the affected diseases. Moreover, the role of spines is mentioned in the development of specific therapies

### 4.1. Neurodegenerative Diseases: Alzheimer’s and Parkinson’s Diseases

In most patients neurodegenerative diseases develop progressively, with participation of synaptic defects already mentioned in healthy patients upon aging. Investigation of synapses, particularly of the spines, can therefore be very relevant to the understanding of the diseases and for the development of their therapy. Lesion examples have been anticipated in the previous section [32,77,82]. Here attention is focused on specific aspects of these diseases.

Alzheimer’s disease is known to include numerous severe symptoms, dependent on aggregation of critical proteins, lipid metabolism and immunity. Synaptic alterations induce accumulation of pre-synaptic vesicles, with ensuing defects of plasticity, learning and memory [86]. Several studies, however, have demonstrated that, in patients already showing severe lesions, cognition can be slowly recovered. Such cognitive resilience processes are due to changes in spine density and morphology [87,88]. This possibility has been strengthened by recent demonstration that remodeling of spine structures is a plausible mechanism to protect synapses by cognitive resilience [88,89].

One of the critical protein alterations widely associated with Alzheimer’s disease is highly phosphorylated tau. Upon detaching from microtubules, such protein is redistributed throughout the cytoplasm, including also the spines [90,91]. The mislocalization of tau, a contributor to spine defects, does not occur only in Alzheimer’s but also in various other neurodegenerative diseases known as tauopathies [92,93]. The convergence of tau-dependent mechanisms and processes in different disease models suggests that the protein plays critical roles. At present, therefore, tau is widely considered promising to develop new and effective treatments in the therapy of neurodegenerative diseases [93].

Parkinson’s disease is caused by the degeneration of dopaminergic neurons in the substantia nigra; this is followed, in striatal spiny neurons, by secondary dendritic pruning, and then the alteration of spines up to their loss. Therapeutic administration of dopamine has been shown to induce the recovery of spines and of long-term motor complications. Development of Parkinson’s disease is therefore accompanied by an abnormality of the mechanisms that control growth and function of synapses [94,95,96].

### 4.2. Autism

Following its first description in 1943, the definition of autism underwent constant revision. The revealed heterogeneity of the identified subgroups led to the comprehensive definition of the disease as autism spectrum disorders (ASD), which is widely accepted. At present many forms of the disease are known to be due to disruptive de novo mutations of an interconnected network containing genes of chromatin remodeling, synapses and Wnt/beta-catenin signaling [97,98]. One of such groups of ASD involves neuronal changes in post-synaptic connectivity. At the subcellular level these alterations correlate with molecular changes in the spine proteome, with alterations of the copy number, topography, and molecular components involved in spine recognition and adhesion. Ensuing effects are short lifetimes of the synapse and compensatory increases in synaptic connections [98]. Here I report two examples of the ASD form dependent on defective spines.

The first example of excitatory spine dependence of ASD, reported a few years ago, is attributed not to a protein’s specificity of these structures but to a defect of their regulation by the actin cytoskeleton [15,19,37,99]. The second example deals with disruptions of CNT4, a protein of the synaptic adhesion contactin superfamily, which in models from humans and mice has been shown to be associated with the risk of autism. In mice this defect is accompanied by a decrease in the excitatory synapses due to defects of the post-synaptic activity, dependent on reduced density of spines. It appears, therefore, that CNT4 is essential for appropriate spine formation. The ensuing defects of synaptic activity are serious risks of autism [100]. The identification of spines/synapses and other mechanisms involved in various forms of CNT4 pathology could promote studies of therapy and identification of their targets in diseases.

## 5. Conclusions

This review is focused on the two types of post-synaptic structures: the flat/indented dendritic synapses, predominant in inhibitory synapses; and the spine synapses, present in almost all excitatory synapses. The variable morphological, electrical and chemical properties of dendrites and their post-synaptic activity enable a spectrum of local and long-range signaling, with a key role in the relationships among neurons and in the evolution of their functions. In other words, diversity in dendritic signaling allows individual neurons to carry out specialized functions within their respective networks.

Although significantly distinct in many respects, the two forms of post-synaptic structures share part of their important properties, including PSD, actin cytoskeleton, and membrane trafficking. Because of their narrow neck (50–400 nm in diameter) and the slow diffusion of their cytoplasmic molecules, however, the spine heads function as partially independent compartments. Thus, dynamic changes in composition, mobility, and signaling of molecules contained within spines, dependent on the diffusional properties of proteins within small compartments, are potentially relevant in many post-synaptic functions. However, post-synaptic responses to single pre-synaptic events are usually small and transient. In order to reach the action potential threshold, numerous responses inducing depolarization need to be integrated. For this, adjacent spines are frequently synchronized in spontaneously active networks, thereby forming dendritic foci that receive locally convergent inputs from pre-synaptic cell assemblies. Among examples dependent on integrated signaling several processes can be mentioned: the neuronal ability to undergo remodeling during morphogenesis; the mechanisms by which synaptic activity sculpts the dendritic synapses and, especially, the spines; the activity-dependence of plasticity; and structural and functional changes induced during learning and memory.

Up to a few decades ago, knowledge about post-synaptic events was still limited. Much exciting progress reported in the present review has been possible because of the recent development of innovative research technologies, such as super resolution microscopy, the implementation of biosensors, high-resolution imaging, fluorescence correlations, and others. New techniques applied in parallel to those employed traditionally, as well as combinations of new techniques, have been employed for the recognition and characterization of important aspects of synapses. Critical questions, however, remain partially unexplained. Therefore, further developments are expected over the next few years.

## Data Availability

The data of this review will be available to all scientists of scientific interest.
